# Second opinions in medical oncology

**DOI:** 10.1186/s12904-020-00619-9

**Published:** 2020-07-21

**Authors:** Ian Olver, Mariko Carey, Jamie Bryant, Allison Boyes, Tiffany Evans, Rob Sanson-Fisher

**Affiliations:** 1 Faculty of Health and Medical Sciences, University of Adelaide, Adelaide, SA Australia; 2 Health Behavior Research Collaborative, School of Medicine and Public Health, Faculty of Health and Medicine, University of Newcastle, Callaghan, NSW Australia; 3 Priority Research Centre for Health Behavior, University of Newcastle, Callaghan, NSW Australia; 4 Hunter Medical Research Institute, New Lambton Heights, NSW Australia

**Keywords:** Cancer, Patient-centred care, Quality of care, Second opinions, Referrals, Consultation

## Abstract

**Background:**

The current study aimed to further our understanding of second opinions among medical oncology patients by examining the proportion of patients who sought a second opinion about their cancer treatment, and why.

**Methods:**

The study was conducted between 2013 and 2015 in three medical oncology clinics located in public hospitals in Australia: in metropolitan New South Wales, metropolitan Queensland, and in Tasmania.

Those patients who provide written informed consent were asked to complete a brief paper and pencil survey in the clinic containing questions on sociodemographic, disease and treatment characteristics. Approximately 1 month later, participants were mailed a second paper and pencil survey which contained questions about whether they had sought a second opinion and their motivation for doing so. Non-responders were followed up by letter at 3 and 6 weeks.

**Results:**

Of 823 patients screened for eligibility, 698 eligible patients, 612 provided consent.

Of those who consented, 355 completed both the initial survey and the second survey and were included in the analyses. Of the 57 patients who sought a second opinion, the most frequent reasons given for doing so were the need for reassurance (49.1%) and the need to consider the range of treatment options (41.8%).

Of the 297 (83.6%) participants who did not seek a second opinion, the main reason was confidence in the first doctor (88.7%). Only 3.1% patients did not know that they could ask for a second opinion. Occasionally the doctor will initiate the referral for a second opinion.

**Conclusions:**

Our study suggests that a minority of cancer patients seek a second opinion at some phase during their care. Most did so for reassurance or to ensure that they had covered all of the treatment options and not because of discomfort or distrust of their treating doctor.

Few patients reported a lack of awareness of second opinions. This suggests that second opinions form part of a patient-centred approach to information provision about care options. Whether the second opinion improves the quality of care or indeed outcomes has been difficult to demonstrate.

## Background

A second opinion is defined as seeking an independent opinion on either diagnosis or treatment by an expert in the same field as the specialist who gave the initial opinion [1]. A second opinion can be sought by the patient, a specialist, a health institution or insurer.

In contrast to the large literature on doctor-initiated second opinions, comparatively less attention has been given to patient-initiated second opinions. This is surprising as the principles of patient-centred care emphasise the need to inform patients about their options and to provide care that is responsive to their needs, values and preferences [2]. Therefore, ensuring that patients know that they have the right to seek a second opinion, and supporting patients to seek a second opinion where they express a preference for this, aligns with the ethos of patient-centred care. Consumers have also recommended that information on the ‘possibility of a second opinion’ be considered an indicator of patient-centred care [3].

A review of 20 quantitative studies of patients with cancer seeking second opinions found that the rates of seeking second opinions ranged between 1 and 88% [1]. Patients with higher levels of education were more likely to seek second opinions than other patients [1]. Another review of seven studies of patient-initiated second opinions found that while most second opinions confirmed the initial diagnosis, between 10 and 60% of second opinions resulted to a change in diagnosis, management or prognosis [4].

The majority of past studies on second opinions have been conducted in the USA [1]. Given differences in health care systems, results are unlikely to be generalizable to countries such as Australia, which has a universal health care system with oncology care freely available via public hospitals and primary care. One prior Australian study which examined the prevalence of patient-initiated second opinions among oncology patients in Australia was a single institution study conducted between 2006 to 2008 by Tattersall et al. [5]. They found that 6.5% of 1892 newly diagnosed oncology patients sought a second opinion. The most frequent reasons given for seeking a second opinion were to seek more information or just gain reassurance about the diagnosis and treatment. A second smaller Australian single institution study found that 33% of 52 patients sought a second medical opinion, most commonly because of communication issues with their first doctor [6].

The current study was part of larger study investigating the role of individual patient, social support and treatment centre variables in the psychosocial outcomes of cancer patients form the patient, multidisciplinary team and administrative perspective. A literature review identified the variables and the resulting patient questionnaires were pilot tested on a patient group. We aimed to further our understanding of second opinions among Australian medical oncology patients by examining the proportion of patients who sought a second opinion about their cancer treatment, and the reasons for seeking or not seeking a second opinion.

The topics align with published papers on second opinion and with the study clinicians’ experiences.

## Methods

The study was conducted in three medical oncology clinics located in public hospitals in Australia: one was located in metropolitan New South Wales, another in metropolitan Queensland, and the other in Tasmania. Approval was gained from University of Newcastle Human Research Ethics Committee as well as from each institution. The study was conducted between 2013 and 2015.

Patients were eligible if they were aged 18 or older, had a confirmed diagnosis of cancer and were attending a participating clinic. Those who were attending the clinic for the first time or who were too unwell to complete the survey were excluded.

A research assistant provided written information about the study and sought informed consent from eligible patients who presented to participating clinics. Those who consented were asked to complete a brief paper and pencil survey in the clinic containing questions on sociodemographic, disease and treatment characteristics. Approximately 1 month later, participants were mailed a second paper and pencil survey which contained questions about whether they had sought a second opinion and their motivation for doing so. Non-responders were followed up by letter at 3 and 6 weeks.

### Measures

#### Demographic characteristics

Age, gender, education, marital status, living situation, employment status, private health insurance status, concession card status was sought by patient self-report.

#### Reasons for seeking a second opinion

Respondents were asked whether they had ever consulted another doctor for a second opinion about their cancer treatment. Those who responded yes were asked, “Why did you seek a second opinion?” Response options included: My family and/or friends asked me to; I needed more opportunity to consider and discuss my options, I needed to be reassured about the best option; I didn’t understand the information or treatment choices the first doctor gave me; I did not trust or agree with the first doctor’s opinion; The first doctor didn’t seem to understand my situation or concerns; I did not feel comfortable with my first doctor; or other.

#### Reasons for not seeking a second opinion

Those who indicated that they had not sought a second opinion were asked why. Response options included: I was confident in my first doctor’s opinion; I didn’t want to upset/ annoy my doctor; I didn’t know which doctor to see; I felt rushed into treatment; I just wanted to start treatment as soon as possible; I didn’t have enough time; I didn’t have enough money; I didn’t know I could get a second opinion; or “other”.

### Statistical methods

Statistical analyses were programmed using SAS v9.4 (SAS Institute, Cary, North Carolina, USA).

Characteristics of the sample are presented as frequencies and percentages for categorical demographic variables and means (standard deviation) and medians (range) for continuous variables.

The results of questions are presented as frequencies and as percentages with 95% confidence intervals estimated by accounting for clustering by treatment center and using the delete-1 jackknife method.

## Results

Of 823 patients screened for eligibility, 125 were ineligible due to one or more of the following factors: attending the clinic for the first time (*n* = 27); no confirmed cancer diagnosis (*n* = 1); non-English speaking (*n* = 70); too unwell (*n* = 6); unable to provide informed consent (*n* = 2), unable to complete the survey independently (*n* = 7), or other reasons (*n* = 12). Of the remaining 698 eligible patients, 612 (88%) provided consent.

Of those who consented, 355 completed both the initial survey and the second survey and were included in the analyses. Comparing those who completed both surveys to those who only completed the baseline survey, the non-completers were more often male (57% compared to 45% who completed, *p* < 0.051) and older (75% over 60 years compared to 46% for completers, *p* = 0.002) Of the patient characteristics, the age range reflects cancer incidence with over half of the participants aged over 60 years, and there was a good spread across the three treatment centres (Table 1).

**Table 1:** Sample demographic

Variable	Characteristic	Total (***N*** = 355)
Gender	Male	159 (45%)
	Female	196 (55%)
Age category	18–40	21 (6.0%)
	41–60	140 (40%)
	61+	190 (54%)
Age	mean (SD)	61.13 (11.68)
	median (min, max)	62.33 (26.99, 100.07)
Married	Married or partner	243 (69%)
	Single, divorced, separated or widowed	110 (31%)
Education	HS or below	151 (43%)
	Vocational training, University or other	199 (57%)
Health insured	Yes	163 (46%)
	No	189 (54%)
Concession	Yes	189 (54%)
	No	164 (46%)
Living with	With Others	279 (79%)
	Alone	74 (21%)
Cancer Type	Colorectal	79 (23%)
	Breast	99 (28%)
	Haematology	13 (3.7%)
	Prostate	21 (6.0%)
	Lung	20 (5.7%)
	Melanoma	12 (3.4%)
	More than one type	98 (28%)
	Other	7 (2.0%)
Number of patients at each treatment centre	101	127 (36%)
	208	120 (34%)
	616	108 (30%)

Of the 57 (16.1%; 95% CI 6.8 to 25.4%) patients who sought a second opinion, the most frequent reasons given for doing so were the need for reassurance (49.1%) and the need to consider the range of treatment options (41.8%) (Table 2).

**Table 2:** Reason for seeking a second opinion (*n* = 57). Frequencies and percentages with confidence intervals

Reason for second opinion	Response	Frequency	Percent	Confidence interval for percentage
Needed reassurance	Yes	27	49.1	(21.0, 77.2)
Needed To Consider Options	Yes	23	41.8	(15.3, 68.3)
Family and Friends	Yes	14	25.5	(0.0, 53.4)
Wasn’t Comfortable with First Dr	Yes	9	16.4	(1.8, 30.9)
Didn’t Trust First Dr	Yes	8	14.5	(0.0, 60.2)
First Dr. Didn’t Understand My Concerns	Yes	5	9.1	(0.0, 22.2)
Didn’t Understand Treatment Choices	Yes	4	7.3	(2.4, 12.1)
Consulted A Second Doctor To Get Access To A Clinical Trial	Yes	2	3.5	(0.0, 11.6)
Second Opinion In Order To Access Treatment Faster	Yes	2	3.5	(0.0, 11.6)
Other/Unclear	Yes	3	5.5	(0.0, 16.8)
Recommended by 1st Doctor	Yes	2	3.5	(0.0, 18.0)

Of the 297 (83.6%) participants who did not seek a second opinion, the main reason was confidence in the first doctor (88.7%). Only 3.1% patients did not know that they could ask for a second opinion (Table 3). Occasionally the doctor will initiate the referral for a second opinion (Table 3).

**Table 3:** Reason for not seeking a second opinion (*n* = 297). Frequencies and percentages with confidence intervals

Reason for no second opinion	Response	Frequency	Percent	Confidence interval for percentage
Confident With First Dr	Yes	259	88.7	(77.9, 99.5)
Not Enough Money	Yes	73	25.0	(19.5, 30.5)
Didn’t Know Which Dr. To See	Yes	12	4.1	(0.0, 8.3)
Wanted to Start Treatment ASAP	Yes	10	3.4	(0.0, 9.5)
Didn’t Know I Could Get a Second Opinion	Yes	9	3.1	(0.0, 9.6)
Not Enough Time	Yes	6	2.1	(0.0, 11.0)
Didn’t Want to Upset Dr	Yes	4	1.4	(0.0, 3.0)
Felt Rushed Into Treatment	Yes	4	1.4	(0.0, 9.6)
Didn’t Feel It Was Necessary	Yes	2	0.7	(0.0, 2.1)
Other	Yes	4	1.4	(0.0, 5.5)

Four participants reported ‘other reasons’ including: “I had enough info from my own investigating to make a decision”, “I was overwhelmed with diagnosis when with doctor;” and “too late, I feel, to start again.” One participant reported that their situation had been discussed at the multidisciplinary team meeting, and so perceived “I’ve had a lot of Drs. Opinion”

A univariable and multivariable logistic regression analysis to assess association of the age, gender, stage, education level and those with health insurance with seeking a second opinion. Having health insurance was associated with seeking a second opinion, after adjusting for age, education, and gender; the odds of seeking a second opinion were two times higher for those with health insurance compared to those without health insurance (95%CI 1.1 to 3.7; *p* = 0.02). The other variables were not associated with seeking a second opinion.

## Discussion

Our study found that 16.1% of participants had sought a second opinion about their cancer treatment. This is between the rates reported In Australia by Tattersall’s and Philip’s study [5, 6]. This most likely reflects methodological differences in that our survey asked participants if they had ever sought a second opinion in relation to their cancer treatment; while the Tattersall study [5] examined second opinions sought at a specific point in time.

The study findings that the main reasons for cancer patients seeking second opinions were reassurance and to seek information to ensure that they had been presented with all of their options reflect previous studies. A review of 13 studies revealed that the two most important topics in seeking second opinions were treatment options and prognosis [7]. In the Australian study by Tattersall et al., 64 of 77 patients who returned questionnaires had received new information from their second opinions [5]. They also felt more confident after receiving a second opinion, particularly if they had expressed dissatisfaction with their initial doctor. Likewise, a group of prostate cancer patients wanted more information but also wanted to see whom they regarded as the best urologist [8]. While losing time was an issue for not seeking second opinions for up to 18% in other studies, only 3.4% of patients in our study wanted to commence treatment as soon as possible [9].

Of 1901 patients consulting medical oncologists early stage breast cancer patients 9.8% sought a second opinion. In addition to higher education and use of internet support groups, uncertain results on genomic testing predicted seeking a second opinion. This may indicate that demand of r seconds opinions may increase with the introduction of more complex genomic tests to guide treatment decisions [10].

The proportion of participants who reported that they did not know they could ask for a second opinion was low in our study. Patients know about second opinions either from their doctors, government agencies or other information forums or consumer organisations [1, 7, 11]. Many patients also gain extra information from friends, relatives and the internet [5, 7].. It has been suggested that patients may feel reassured about their care if they knew that treatment guidelines existed and that their case had been discussed by a multidisciplinary team [5, 7]. However two studies found that knowledge that their treatment plan came from a multidisciplinary team had no impact on patients’ decision to seek a second opinion [5, 12].

The rate of dissatisfaction with the doctor giving the primary opinion was lower in our study (16.4%) than in multiple other studies where it has been reported by around 30% of participants [6, 13]. In a study involving prostate cancer patients, seeking a second opinion was not associated with accepting definitive treatment [8]. This raises the possibility of patients shopping for opinions that align with their wishes. Dissatisfaction is often due to communication issues between doctors and patients [6]. It has been suggested that satisfaction with a second opinion may involve patient factors where a changing emotional state over time may make them readier to hear information as time passes [6].

Trust in the physician was a major reason for patients not seeking a second opinion [9, 14]. Moreover some patients saw seeking a second opinion as possibly affecting the relationship with the clinician who gave the initial opinion [6, 15]. Many oncologists have no issues with their patients seeking second opinions and often associate seeking a second opinion with the patient having greater information and support needs [6].

The biggest issue for future studies is whether referral for a second clinical opinion actually improves quality of care and clinical outcomes, since, unlike with diagnostic studies this has been difficult to demonstrate [7, 16]. Some studies have shown doctors second opinions are influenced by the initial opinion and those giving subsequent opinions are certainly aware of the medico-legal consequences of their consultation [6]. If consultations are by multi-disciplinary teams and follow evidence -based guidelines there may be little difference between treatments, particularly if the major issue for seeking the second opinion was a communication problem. This would explain why knowledge of their care had been discussed by a multidisciplinary team had no impact on patients’ second opinion seeking.

The limitations of the generalisability of the study findings include the exclusion of participants who did not speak English and recruitment from three metropolitan hospitals. Recall bias from the participants who were diagnosed more than 1 year ago may result in a slight underestimation of the frequency of obtaining a second opinion.

It would be worth exploring further whether patients having easier access to evidence-based guidelines may prove reassuring. That was part of motivation for web-based wiki guidelines in cancer that could be widely disseminated an easily accessed [17]. Issues to be explored in future studies of second opinions include collection of data on whether the second opinion changed the treatment plan proposed by the first consultation. Secondly, those receiving a second opinion could have their cancer outcomes compared to a matched group who did not request a second opinion with cancer outcomes of survival and progression free survival assessed. There is not a good economic analysis of the cost benefit of second opinions. Finally, a longitudinal study of patient and provider satisfaction would help determine the longer-term impact of a second opinion. It is also important to ascertain further patient characteristics which may predict the likelihood of seeking a second opinion.

## Conclusions

Our study suggests that a minority of cancer patients seek a second opinion at some phase during their care. Most did so for reassurance or to ensure that they had covered all of the treatment options and only 14.5% because of discomfort or distrust of their treating doctor.

Few patients reported a lack of awareness of second opinions. This suggests that second opinions form part of a patient-centred approach to information provision about care options. Whether the second opinion improves the quality of care or indeed outcomes has been difficult to demonstrate.

## Data Availability

The datasets during and/or analysed during the current study available from the corresponding author on reasonable request.

## Abbreviations

%: Per centage
CI: Confidence Interval
n: Number
p: Probability value
SAS: Statistical Analysis System
USA: United States of America
